# Cutaneous immune-related adverse events to immune checkpoint inhibitors: from underlying immunological mechanisms to multi-omics prediction

**DOI:** 10.3389/fimmu.2023.1207544

**Published:** 2023-06-22

**Authors:** Ting Cao, Xuyang Zhou, Xingbiao Wu, Ying Zou

**Affiliations:** Allergic Dermatoses Clinical Center, Shanghai Skin Disease Hospital, Tongji University School of Medicine, Shanghai, China

**Keywords:** immune checkpoint inhibitors, cutaneous immune-related adverse events, multi-omics, biomarkers, cutaneous

## Abstract

The development of immune checkpoint inhibitors (ICIs) has dramatically altered the landscape of therapy for multiple malignancies, including urothelial carcinoma, non-small cell lung cancer, melanoma and gastric cancer. As part of their anti-tumor properties, ICIs can enhance susceptibility to inflammatory side effects known as immune-related adverse events (irAEs), in which the skin is one of the most commonly and rapidly affected organs. Although numerous questions still remain unanswered, multi-omics technologies have shed light into immunological mechanisms, as well as the correlation between ICI-induced activation of immune systems and the incidence of cirAE (cutaneous irAEs). Therefore, we reviewed integrated biological layers of omics studies combined with clinical data for the prediction biomarkers of cirAEs based on skin pathogenesis. Here, we provide an overview of a spectrum of dermatological irAEs, discuss the pathogenesis of this “off-tumor toxicity” during ICI treatment, and summarize recently investigated biomarkers that may have predictive value for cirAEs *via* multi-omics approach. Finally, we demonstrate the prognostic significance of cirAEs for immune checkpoint blockades.

## 1 Introduction

The development of immune checkpoint inhibitors (ICIs), such as monoclonal antibodies targeting programmed death-1 (PD-1)/programmed death-ligand 1 (PD-L1) and cytotoxic T-lymphocyte-associated antigen 4 (CTLA-4), has dramatically changed the landscape of therapy for multiple malignancies. ICIs represent one type of immune therapy for cancer, among other options such as, surgery, chemotherapy, radiotherapy, targeted therapy and immune therapy. In contrast to other therapies that use toxic chemical or physical agents to kill tumors, immunotherapy aims to harness the immune response. Immunotherapy is premised based on the theory that the immune system should be able to eliminate tumors, but the tumors ‘escape’ by some mechanisms, termed ‘immunoediting’ (1, 2). Accordingly, immunotherapy kills tumors by enhancing the anti-tumor ability of the immune system or by inhibiting tumor immunoediting. It has been established that checkpoints are some regulators of immune response, and tumors could specifically stimulate some negative checkpoints to suppress the immune response, thus escaping (3). Therefore, the immune system may be used to target tumors by inhibiting negative checkpoints, such as CTLA-4 and PD-1/PD-L1.

The intervention of immune homeostasis by ICIs can enhance the anti-tumor function of the immune system, while also leading to some adverse effects resulting from the systemic immune overactivation (**Table 1**). These unwanted effects are often termed immune-related adverse events (irAEs). Among all the related organs and systems, the skin is one of the most common targets, of which cutaneous irAEs (cirAEs) are often the first to manifest (5). Since the suppressive effects of checkpoints on the immune response are inhibited by ICIs, lymphocytes become over-activated (22, 23), pro-inflammatory cytokines are abundantly released (24, 25), and immune tolerance is destroyed (26, 27), all of which may contribute to the irAEs. These adverse events present a challenge for cancer patients receiving ICIs and can even force them to withdraw from ICI therapy. However, the precise mechanism of irAE remains unknown, and treatment primarily comprises immunosuppressants, such as glucocorticoids (28). Although there is scant evidence showing that the application of immunosuppressors can offset the anti-tumor effect of ICIs (29), the development of new adverse events (e.g., opportunistic infection, hyperglycemia, fluid retention, anxiety, and osteoporosis) should not be ignored over the long term (30, 31).

**Table 1:** Common cirAEs.

cirAEs	Manifestations	Immune checkpoints	Ref.
Pruritus	Inflamed skin and scratch marks	PD-1/PD-L1, CTLA-4	(4–7)
Maculopapular rash	Faint erythematous macules and papules coalescing into plaques	PD-1/PD-L1, CTLA-4	(5, 8, 9)
Bullous pemphigoid (BP)	Large, fluid-filled blisters located in between skin folding or creases of skin	PD-1/PD-L1, CTLA-4	(10–12)
Vitiligo	Patchy loss of skin color, premature whitening or graying of the hair,	PD-1/PD-L1, CTLA-4	(6, 13, 14)
Psoriasiform	Patchy rash varying in color, small scaling spots, dry and cracked skin	PD-1/PD-L1, CTLA-4	(13, 15)
Eczema	Dry and cracked skin, itchiness, rash on swollen skin	PD-1/PD-L1, CTLA-4	(5, 16, 17)
Stevens Johnson Syndrome (SJS)	Painful raw areas called erosions that resemble a severe hot-water burn	PD-1/PD-L1, CTLA-4	(18)
Toxic epidermal necrolysis (TEN)	Widespread skin pain, spreading rash, blisters and large areas of peeling skin, sores, swelling and crusting on the mucous membranes, including the mouth, eyes and vagina	PD-1/PD-L1, CTLA-4	(19, 20)
Drug reaction with eosinophilia and systemic symptoms (DRESS)	An extensive mucocutaneous rash, accompanied by fever, lymphadenopathy, hepatitis, hematologic abnormalities with eosinophilia and atypical lymphocytes	PD-1/PD-L1, CTLA-4	(6, 21)

Since cirAEs occur often and early, influencing the life quality of patients, which reduces patient compliance to ICIs, the treatment of which also gives rise to a series of new problems, there is an urgent need to identify predictive biomarkers of cirAEs. Several risk factors have been identified by epidemiological investigation, and the serum levels of several molecules in patients suffering from irAEs have been found to exhibit significant differences compared to those without irAEs (**Table 2**). However, none of these biomarkers have shown satisfying prediction efficacy, which may be due to the heterogeneity and complex mechanisms of irAEs. The recent advent of multi-omics, a combined technology including genomics, transcriptomics, proteomics and metabolomics, has been associated with substantial progress for revealing the mechanism and predicting irAEs. Analyzing the genome helps us to find mutations that are responsible for ICI-resistance and irAEs, thus contributing to uncovering the mechanism of irAEs and predicting the risk. Regarding to the heterogeneity, transcriptomics deals with the distinct expression of genes, providing a context-dependent understanding of what actually occur in the anti-tumor immunity, and proteomics provides functional insight into genomics. Moreover, since the metabolic reprogramming is a hallmark of cancers, which is associated with the tumorigenesis, progression, metastasis and drug-resistance, the screening for metabolomics reveals the current condition or status, helping to determine whether the tumors are responsive to ICIs and whether the immune homeostasis is disturbed to elicit adverse events (51–53). From a systemic biology perspective, a macroscopic immune network has gradually been uncovered and additional molecules that were previously unknown have been identified as biomarkers for the prediction of both anti-tumor efficacy and irAEs.

**Table 2:** Some current biomarkers of irAEs.

Category	Biomarker	Specific irAE	Specific cancer type
Serum factors	IL-6 (32–34)	Non-specific	Non-specific
	IL-17 (25, 35)	ICIs-induced colitis,	Melanoma
	C reaction protein (36–38)	Non-specific	RCC, NSCLC
	Preexisting auto-antibody (39–41)	Endocrine irAEs	Non-specific
	Serum neurofilament light chain (42)	Neuro irAEs	Non-specific
Cells	Neutrophil to lymphocyte ratio (43)	Non-specific	Non-specific
	Platelet-to-lymphocyte ratio (44)	Non-specific	NSCLC
	IgG4^+^/PD-1^+^ MFI ratio (45)	Non-specific	Non-specific
	Tumor Infiltrating Lymphocytes (46)	Cutaneous irAEs	Melanoma
Others	TMB (47)	Non-specific	Non-specific
	Circulating tumor DNA (48, 49)	Non-specific	Non-specific
	Indoleamine 2,3-dioxygenase 1 (50)	Immune-mediated hepatotoxicity	Non-specific

RCC, renal cell carcinoma; NSCLC, non-small cell lung cancer; IL-6, interleukin-6; IL-17, interleukin-17; TMB, tumor mutation burden; MFI, mean fluorescence intensity.

## 2 Epidemiology and clinical manifestations

Cutaneous irAEs is one of the most common types of irAEs, with regards to morbidity. cirAEs arise in as many as 34% of patients receiving anti-PD-1/PD-L1 therapy and about 43% −45% of those on CTLA-4 inhibitors (54). The incidence of cirAEs varies among the patients suffering from different types of cancers, and even different pathological subtypes and different stages of certain cancers (55). Distinct types of ICIs can also lead to distinct incidence of cirAEs (56). Nevertheless, cirAEs commonly manifest as maculopapular rash, psoriasiform rash, bullous pemphigoid (BP), vitiligo, pruritus, eczema, and Stevens-Johnson syndrome (SJS), toxic epidermal necrolysis (TEN), drug reaction with eosinophilia and systemic symptoms (DRESS) less commonly (13). Cutaneous irAEs occur early, with the time to onset ranging from 3 to 4 weeks (57), compared to 12 weeks in the endocrine gland (58) and 22.2 weeks in the gastrointestinal tract (59). Therefore, for the common and early onset and suffering manifestations, there is an urgent need to investigate cirAEs, uncovering its mechanism, and identifying its predictive biomarkers. Such information will serve to relieve the pain of patients as well as contribute to cancer therapy.

## 3 Mechanisms

**Figure 1:**
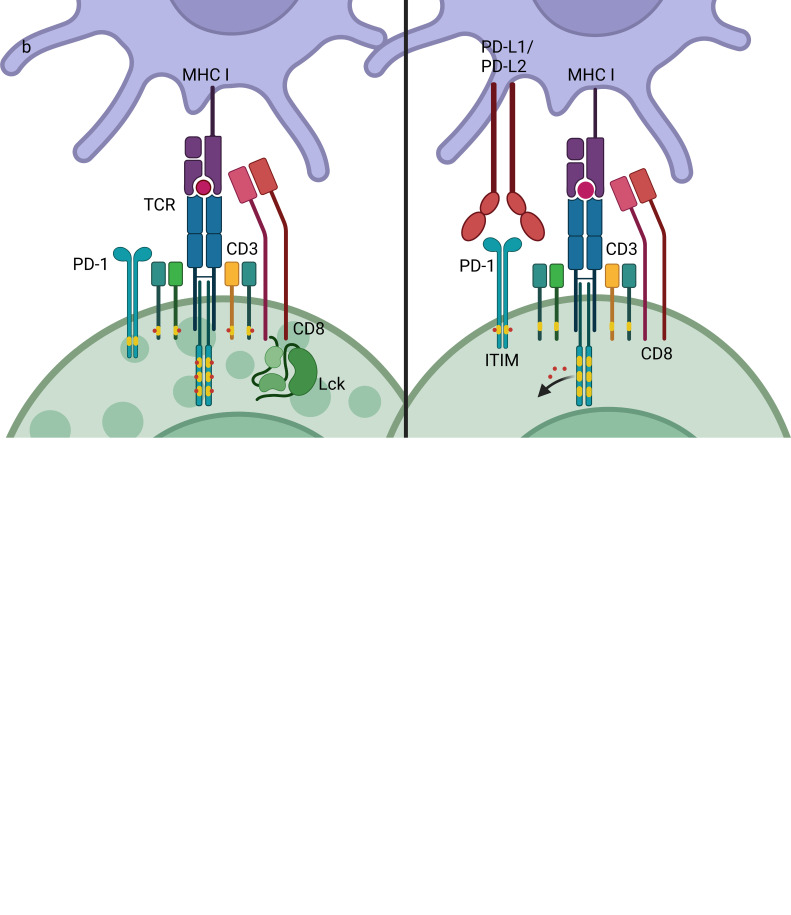
Mechanisms of CTLA-4 and PD-1/PD-L1.CTLA-4 and PD-1 are both negative checkpoints that inhibit the activation of lymphocytes. **(A)** T cells can be activated by APCs by pMHC-TCR (signal 1) and co-stimulatory molecules such as B7-CD28 (signal 2). CTLA-4 is competitive inhibitor of CD28, which has higher affinity to B7, thus inhibiting the co-stimulatory signaling in T cells activation. **(B)** PD-1 is an inhibitory receptor that contains an ITIM or ITSM, which mediate dephosphorylation reaction once activated, thus stopping the activation signaling transduction. CTLA-4, cytotoxic T-lymphocyte-associated antigen 4; PD-1, programmed death-1; PD-L1, programmed death-ligand 1; MHC, major histocompatibility complex; TCR, T cell receptor; ITIM, immunoreceptor tyrosine-based inhibition motif; ITSM, immunoreceptor tyrosine-based switch motif.

ICIs are agents that block the interaction between checkpoints and the associated ligands and thereby block the subsequent intracellular signaling. The most commonly used ICIs target PD-1/PD-L1, with others targeting of CTLA-4, Tim-3, and LAG-3. Although both CTLA-4 and PD-1/PD-L1 are negative checkpoints, they play different roles in regulating the immune response, thus leading to different adverse events once blocked. CTLA-4 is a competitive inhibitor of CD28, a co-stimulatory signal receptor that is essential for T cell activation (**Figure 1A**). CTLA-4 is considered to be the most important negative checkpoint, as murine animals lacking CTLA-4 will die at an early age due to severe lymphoproliferation (60). Moreover, regulatory T cells (Treg) also function *via* CTLA-4 expression, competitively binding to B7 expressed on antigen presenting cells (APCs), which blocks its co-stimulatory effects on naïve T cells (61). PD-1 is one of the inhibitory receptors that contain an immunoreceptor tyrosine-based inhibition motif (ITIM) or the related immunoreceptor tyrosine-based switch motif (ITSM), which could remove the phosphates once activated and thereby inhibit the signaling (**Figure 1B**). PD-1 is able to bind with PD-L1 and PD-L2, which are constitutively expressed by a variety of cells and inductively expressed on APCs during inflammation, respectively (62, 63). Regulating the expression of PD-1 can control the intensity of the immune response, as pro-inflammatory cytokines have been shown to down-regulate PD-1 expression and murine models lacking PD-1 tend to develop auto-immune diseases (64). Therefore, various checkpoints substantially contribute to the regulation of the immune response and tolerance. Medications that affect checkpoints may lead to a disorder in immune homeostasis.

Having established a macroscopic overview of the action of ICIs in the immune response, we will now discuss the mechanism of irAEs, especially cirAEs. In general, irAEs are primarily induced by the overactivation of the immune response due to a blockades of negative regulators, and auto-immune responses are activated as a result. As previously discussed, cirAEs manifest commonly and early, indicating that cirAEs have some distinct characteristics other than the common mechanism of irAEs. Here, we propose that the commensal microbiota and the distinct characteristics of cutaneous immune system may be the issue. Next, we will first demonstrate the role of auto-immune response in irAEs, followed by a discussion of the commensal microbiota and the characteristics of cutaneous immunity. Finally, we will discuss genetic factors.

### 3.1 Autoimmunity

An important mechanism of irAEs is the autoimmune response, which is the immune response that targets self-antigens. Many irAEs are considered to be or appear to mimic autoimmune diseases, including myocarditis (65), diabetes mellitus (66), hypothyroidism (67), pneumonitis (68), rheumatoid arthritis (69), vitiligo (70), BP (10) and psoriasis (71). The association between checkpoints and autoimmunity has previously been confirmed by the Genome-Wide Association Study (GWAS), in which mutations in the CTLA-4 and PD-1/PD-L1 genes were identified to be responsible for several autoimmune diseases, such as Grave’s Disease and systemic lupus erythematosus (SLE) (72, 73). Other studies have also demonstrated that the IL-27-mediated priming of naïve T cells could upregulate the expression of PD-L1, which inhibited the differentiation of CD4^+^ T cells into a Th17 phenotype, thereby exhibiting protection against autoimmune diseases (74). The mechanism of this protection also involved a blockade of the TCR from binding to dendritic cells (DCs) through the interaction of PD-1 and PD-L1 (75). Moreover, PD-L1 were found to be abundantly expressed on pancreatic β-cells to avoid autoimmune attack (76). Therefore, a PD-1/PD-L1 blockade may induce autoimmune diabetes (type 1 diabetes mellitus, T1DM) by destroying this tolerance. *Pdcd1^-/-^Ctla4^+/-^* mice can be used as the model to study ICI-associated myocarditis (ICI-MC) (77). Moreover, the engineered expression of PD-1/PD-L1 have been used for the treatment of several immune diseases, including arthritis, colitis, and T1DM (78, 79). This indispensable role of PD-1/PD-L1 for preventing autoimmunity was also confirmed by a clinical research in a patient with an inherited PD-1 deficiency, diagnosed with T1DM, hypothyroidism, and idiopathic arthritis, who was dying from severe pulmonary autoimmunity (80). In fact, although the mechanism of ‘central tolerance’ eliminates most of the lymphocytes which could be activated by self-antigens, self-reactive lymphocytes always exist in the natural immune repertoire but do not elicit remarkable autoimmune diseases, due partly to the lack of activation signal (also termed as ‘second signal’) and the action of negative checkpoints such as PD-1/PD-L1, termed ‘peripheral tolerance’, thus keeping a balance between preventing infection and preventing autoimmunity. In some contexts, infection is a common trigger of autoimmune diseases because it leads to an abundant release of pro-inflammatory cytokines, which act as activation signals towards self-reactive lymphocytes. The application of ICIs may also activate the autoimmune response by liberating self-reactive lymphocytes from the inhibitory control of negative checkpoints. Therefore, in this manner, irAEs of ICIs often appear to mimic classic autoimmune diseases. Since self-active T cells can induce the autoimmune response *via* two methods, exhibiting direct cytotoxicity and facilitating B cell-induced immune response, auto-antibodies are also involved in the irAEs, which is in line with previous literature. The hypophysitis and diabetes mellitus induced by anti-CTLA-4 and anti-PD-1/PD-L1, respectively, can serve as examples of cases in which auto-antibodies, while undetectable at baseline, developed significantly following treatment with ICIs (64, 81). Another important autoimmune disease is bullous pemphigoid (BP), which is associated with impaired basement membrane zone (BMZ) caused by auto-antibodies targeting BP180 and BP230. Trauma, burn or radiation may elicit BP by destroying the immune barrier which leads to the exposure of self-antigen to self-active lymphocytes, and application of some drugs also results in BP which may due to the immune-modulatory effects of these drugs. ICBs will also give rise to disturbance of immune responses. The increased risk of BP in patients receiving anti-PD-1/PD-L1 has been confirmed by series of clinic researches (11, 13, 82, 83), while the molecular mechanisms still need further researches to elucidate. A depletion of Tregs, which function depending on immune checkpoints, may play a role in the pathogenesis of BP, according to immunopathological results (84).

**Figure 2:**
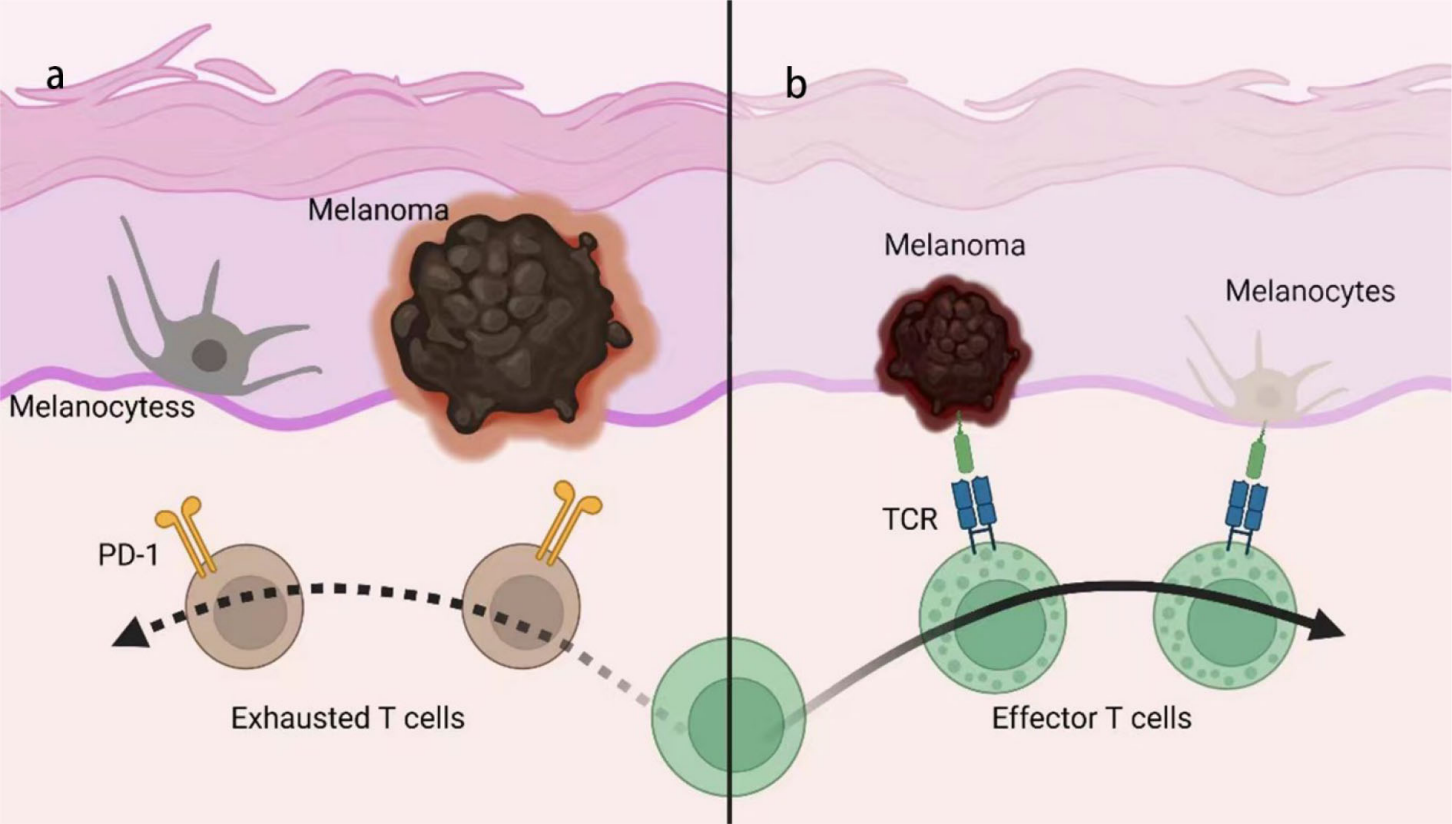
Mechanism of anti-PD-1/PD-L1-induced vitiligo. **(A)** Melanoma and melanocytes exhibit some shared antigen but are protected from immune attack by peripheral tolerance mechanisms, such as PD-1/PD-L1. **(B)** With PD-1/PD-L1 blocked, T cells will be activated by melanoma and initiate immune response targeting both tumors and normal tissue, due to the shared antigen on melanoma and melanocytes, termed ‘cross-activation’. TCR, T cell receptor.

The relevance of anti-tumor efficacy and the irAEs of ICIs also implies the autoimmune mechanism of irAEs. While some studies did not confirm the association between the treatment efficacy and irAEs (29), others did find that the occurrence of irAEs was usually associated with a more robust response to ICI therapy and better prognosis (70, 85–88). GWAS studies also identified an IL-7 variant that can lead to increased irAEs incidence and a concomitant increase in overall survival in melanoma patients (89, 90). The failure of the immune system to eliminate tumors is partially due to a lack of a true ‘onco-antigen’, that is, the tumors do not present distinct antigens that can be recognized by the immune system rather than being tolerated. This is because tumors comprise part of our body and the mechanism of immune tolerance can prevent the body’s response to them. The condition may differ with the application of ICIs, which interferes with immune homeostasis. Several studies have found that severe irAEs were associated with a longer overall survival, even while regarding irAEs as an indicator for predicting the prognosis of patients receiving ICIs. This finding may be partly explained by the fact that ICIs can promote the anti-tumor immune response by inhibiting immune tolerance, as PD-1/PD-L1 plays an important role in mediating the immune tolerance. An example is vitiligo, a common irAE in melanoma patients receiving anti-PD-1 therapy that is caused by an autoimmune attack to melanocytes (**Figure 2**). Research has proposed that this effect is due to the cross-reactivity between T cells directed against tumors and a related antigen expressed in normal tissues (27). Under normal physiological conditions, T cell targeting of antigens expressed on normal melanocytes will not be activated, nor will those that target related antigens expressed on tumors due to the immune tolerance. Therefore, neither the auto-immune response nor the anti-tumor response is intensely elicited. Once tolerance is destroyed by PD-1/PD-L1 blockade, these T cells will be activated by melanoma or by melanocytes, thus attacking melanoma cells as well as normal melanocytes, which may account for the relevance between the efficacy and irAEs of ICIs. Moreover, this theory may help explain why the irAEs vary among the different types of cancers, depending on the cross antigens between tumors and normal cells. Whether the PD-1/PD-L1 blockade could promote anti-tumors immunity *via* decreasing tolerance to tumors requires validation with further research. However, the role of checkpoints in preventing autoimmune diseases and the relationship between the severity of irAEs and better prognosis have been established by previous studies.

### 3.2 Targeting at commensal microbiota

The link between commensal microbiota and response to ICIs for the treatment of cancer has been revealed by a series of studies that investigated the heterogeneity of the patients’ response to ICIs (91–98). Using 16S ribosome RNA gene sequencing, both preclinical and clinical studies have found distinct commensal microbiota between patients who are ICI-responsive and non-responsive (97, 99). There is increased concern that the commensal microbiota and its metabolites have a substantial influence on host homeostasis. For example, the interaction between the microbiota and host can ‘shape’ the immune system of the host. As a result, there is a heterogeneous response to ICIs due to the heterogeneity of the commensal microbiota to some extent. Although the association between the anti-tumor efficacy and the irAEs of ICIs remains conflicting (29, 85, 100–102), some studies have actually found a positive relationship (100–102), indicating that commensal microbiota may play a role in the pathogenesis of irAEs. It has been well established that the colon is the location in which there is the most abundant commensal microbiota, and the skin is another important residence. Epidemiological investigations have shown that irAEs most commonly involve the skin, GI tract, and endocrine system (5, 103). The microbiota abundance and irAE frequency in the skin and GI tract may indicate the potential relevance of commensal microbiota. One study confirmed an association between irAEs and several *Lachnospiraceae* spp. and indicated that the abundance of *Streptococcus* spp. substantially contributes to the distinction of irAEs (91). Additional studies have also supported the association between the commensal microbiota and irAEs (93, 95, 104). Reports researching the impact of PPIs have found that the application of PPIs has also had an impact on irAEs by influencing the microbiome (105, 106). The commensal microbiota profile may be used to predict irAEs (93) and the therapy targeting the commensal microbiota, such as fecal microbiota transplantation (FMT), may be used to cure irAEs (107).

Skin is the first-line barrier to protect the host from microbial invasion while maintaining a peaceful coexistence with resident microbiota (108), along with other barriers, such as the GI tract, respiratory tract and genital tract. A homeostatic state is formed under the complex microbiota-host interaction network and the intensity of immune response is controlled to a ‘set point’, which is suitable for the micro-ecosystem. Immune homeostasis is maintained by many immune regulators, including cytokines, regulatory receptors and regulatory immune cells. The application of ICIs makes great intervention for this control mechanism. Physiologically, within the action of checkpoints, T cells will not become activated to target commensal microbiota due to the lack of activation signals and presence of inhibition signals, plus some regulatory immune cells (e.g., Tregs) and cytokines (e.g., IL-10). Once CTLA-4 or PD-1/PD-L1 is blocked, however, CD4^+^ and CD8^+^ T cells may be activated and induce a subsequent immune response, which can lead to tissue damage. A recent study found that *Staphylococcus epidermidis* could only elicit inflammation in the context of a CTLA-4 blockade, the latter of which resulted in excessive activation of IL-17-producing commensal-specific T cells; thus inducing skin damage (109). Moreover, since the commensal microbiota itself also plays a crucial role in maintaining immunological homeostasis, an inappropriate immune response causes indirect damage by inhibiting commensal microbiota. Research into inflammatory bowel disease (IBD) has revealed that the failure to limit inappropriate inflammation contributes to ulcerative colitis and Crohn’s disease (110–112), and other research about atopic dermatitis (AD) has found that microbiota diversity was decreased in inflamed AD skin (113) and reverted during the treatment and recovery (114). Moreover, epidermal barrier dysfunction represents a key factor associated with the pathogenesis of AD, which can be due to an over-release of pro-inflammatory cytokines and damage-associated molecular patterns (DAMPs) (115), such as that seen in inherited filaggrin deficiency (116). Further research into the influence of ICIs on the cutaneous micro-economy must be conducted. In summary, the cutaneous commensal microbiota is highly involved in the pathogenesis of cirAEs and the immune responses targeting commensal microbiota can lead to tissue damage by both direct and indirect methods, as a result of impaired immune tolerance.

### 3.3 The cutaneous immunity

In this section, we will aim to explore that why the skin is a common and early target of irAEs. The hallmark of the skin from an immunological perspective is the abundance of immune molecules and cells, as well as higher activity of the immune response. The immune profile is determined by and is suitable for the systemic role of the skin as a barrier for the entire body and its corresponding physiological functions. Our internal environment is separated from the external environment by the skin and mucosa, which covers the surface of body and the internal lumen, respectively, such that the skin is exposed to the most direct effects of various of physical, chemical and biological factors. The role of the skin to protect the internal environment from being affected by these disturbing agents, thereby helping to maintain internal homeostasis, so as to increase the active immunity of the skin. Similar to the colon, which also continuously makes contact with a wide range of foreign antigens and may lead to diarrhea, abdominal distension, and abdominal pain if the local immune response is induced (117–120), the skin also continuously faces a multitude of foreign stimuli. Moreover, since the immune responses to these factors can occasionally be senseless and challenging, immune tolerance is of greater significance in the skin compared to other parts of the body. UV radiation will give rise to DNA damage (121, 122), contacting chemicals will also affect, and the commensal microbiota is an obvious source of foreign antigens. Using the immune response induced by UV radiation as an example, keratinocytes could be activated by UV radiation, initiating the formation of NLRP3 inflammasome and releasing IL-1β, which acts as an important pro-inflammatory cytokine (123, 124). By blocking the mechanisms used to limit the immune response, the persistent stimuli of UV radiation can over-activate the immune response in both intensity and duration, leading to cutaneous disorders. Therefore, the skin is a common target for the destruction of immune tolerance by ICIs. Moreover, the immune response in the skin is more readily initiated due to the abundance of the immune cells residing within the skin. Keratinocytes and melanocytes represent two major types of epidermal cells which generate keratin and melanin and act as crucial components of the skin barrier, respectively. In addition, both of these cell types express TLRs and NLRs and can initiate the immune response by secreting pro-inflammatory cytokines and chemokines to activate and recruit other immune cells (125, 126). Another important cell type that can induce an immune response is Langerhans cells (LCs), which can be regarded as a specific type of dendritic cell for its similar antigen-presenting function as a classic DC, whereas recent studies have shown that LCs are resident macrophages in the epidermis (127). Additionally, tissue-resident memory T cells (T_RM_) also contribute substantially with their ability to rapidly recall the immune response by releasing cytokines or exhibiting cytotoxicity (128). In particular, CD8^+^ T_RM_ have been shown to patrol the tissue or function as a local sentinel in both epidermal and dermal layers, providing a rapid and tissue-wide immune response (129). Significantly, there is a constitutive expression of negative checkpoints (e.g., PD-1, LAG-3, and Tim-3) on these T_RM_ in the skin (130, 131). Therefore, the application of ICIs may lead to the over-activation of T_RM_. Taken together, these characteristics of cutaneous immunity indicate that the skin is more susceptible to the adverse events induced by drugs that affect immune homeostasis and the onset of cirAEs occurs swiftly due to the rapid responses of these immune cells.

In addition to inducing a local immune response, the skin is also responsible for transmitting invading signals to the brain; thus, the skin is largely innervated by sensory nerves. The role of inflammation on inducing sensations such as pain and itching is relatively clear (132). It has been shown that some neuropeptides, such as substance P (SP) and vasoactive intestinal peptide (VIP), can activate immune cells (e.g., mast cells), as confirmed by previous studies (133–135). Additionally, several studies have reported highly complex crosstalk or interactions between the immune response and these sensations. Moreover, the experimental application of imiquimod (IMQ) in murine skin could provoke inflammatory lesions that resemble human psoriasis. This effect was found to be blocked pharmacologically or through genetic ablation of nociceptors and could be restored by exogenous IL-23. A subsequent detailed study confirmed that the sensory neurons expressing the ion channels, TRPV1 and Na_V_1.8, could regulate the production of IL-23/IL-17 by interacting with dermal dendritic cells to modulate the local immune response (136). Another subset of neurons expressing MrgprD was shown to inhibit the degranulation of mast cells and limit the cutaneous immune response *via* releasing glutamate. Indeed, the loss of these neurons may lead to immune disorders (137). There is also a subset of macrophages that have been identified to interact with sensory nerves, surveilling and trimming the myelin sheath (138). Due to the complicated interaction between sensory nerves and immune cells, such homeostasis appears to be susceptible to intervention, and some unpleasant sensations (e.g., chronic pain and itchiness) are commonly induced once the immune response is activated, as the manifestation of cirAEs.

### 3.4 Genetics

We have established that the irAEs are closely related to autoimmunity. Despite the unclear precise mechanism, epidemiological investigations have found that autoimmune diseases usually have a strong genetic component, which means some are easier to suffer from these diseases, whereas others are not. Psoriasis is a common disease that cirAEs appear to mimic (also termed ‘psoriasiform rash’) after using PD-1/PD-L1 (139, 140) and is considered to be an autoimmune disease to some extent (141, 142). Previous research has found that in people with parents suffering from psoriasis are easier to suffer from this disease (143), several psoriasis susceptibility genes have been identified, including HLA-Cw6, IL12B, IL23R, and LCE3B/3C (144). T1DM is another example which is also a classic autoimmune disease and one of the most common irAEs. The primary risk factor for β-cell immunity is confirmed as genetic, which mainly occurs in individuals with either HLA-DR3-DQ2 or HLA-DR4-DQ8 haplotypes, or both (145). Alopecia is the most common hair toxicity associated with ICIs and has a phenotype similar to alopecia areata (AA) (13). The GWAS study identified 139 SNPs associated with AA and demonstrated an autoimmune mechanism (146). Genomics-based and recent multi-omics-based approaches have shed light on the research into autoimmune diseases and irAEs. Some of these biomarkers may potentially be applied for the prediction and precise treatment of diseases in the future.

## 4 Prediction of cirAEs

Since cutaneous irAEs occur often and early, patients suffer and are forced to withdraw, and there is an urgent and unmet demand for seeking validated biomarkers to predict cirAEs due to its high morbidity and negative influence on cancer immunotherapy. This task can be conducted by traditional epidemiological methods, such as cross-sectional, cohort, or case-control studies (147–149). The probable biomarkers that have currently been identified are either general characteristics (e.g., age (150, 151), gender (152), and BMI (153)) or common serum molecules based on the current understanding of the immunological mechanisms of cirAEs (e.g., CRP (154), IFN-γ (155)). Regarding to the antigenicity, tumor mutation burden (TMB) (156–158) and microsatellite instability (MSI) (159, 160), which stand for the neoantigen or onco-antigen, are used to predict the efficacy of ICIs and the risk of irAEs. As for the strategies by which tumors suppress the immune response, the expression of PD-L1 was also considered as a predictive biomarker for the responsiveness of ICIs (161). However, these biomarkers do not provide appropriate predictions in clinical practice, and the results of studies identifying these biomarkers also remain conflicting. These discrepancies are partially due to the heterogeneity of the cirAEs. As previously discussed, cirAEs are closely related to cutaneous commensal microbiota and autoimmunity, both of which are highly heterogeneous among the population. Therefore, general characteristics alone may not be sufficient to induce cirAEs and those serum factors often play a common role in several pathways in the immune network, thereby lacking precision. Traditional research methods of molecular biology explore the role of a ‘pathway’ rather than dealing with the ‘network’, ignoring the crosstalk among the pathways which appear to be independent. In contrast, the technology of multi-omics screens for the whole genome, transcriptome, proteome and metabolome, analyzing the whole signaling network in different stages from gene to metabolites. It also helps to study commensal microbiota, which is unavoidable in cirAE research, as discussed previously.

Researchers have identified *LCP1* and *ADPGK* as irAE biomarkers by conducting a comprehensive screening across mRNA, miRNA, lncRNA, protein expression and non-silent gene mutations across 26 cancer types, in which lymphocyte cytosolic protein 1 (*LCP1*), involved in T cell activation, achieved the highest correlation coefficient. The addition of the adenosine diphosphate-dependent glucokinase (*ADPGK*), which can mediate a metabolic shift during T cell activation, to *LCP1*, led to a linear-regression model with the best accuracy among all the bivariate models. These two biomarkers were validated by a subsequent cohort study, which involved 28 cancer patients receiving anti-PD-1/PD-L1 therapy and found higher geometric mean and stronger immunohistochemistry staining in the irAEs group. The area under the receiver-operating characteristic curve (AUC) of *LCP1* and *ADPGK* to predict irAE was 0.78 and 0.78, whereas the combination of *LCP1* and *ADPGK* had a better AUC value at 0.80 (162). Another study established a tri-variate model composed of CDC42EP3-206, TMEM138-211, and IRX3-202 to predict irAEs by combining pharmacovigilance data and pan-cancer transcriptomic information (163). RNA and whole exon sequencing of tumors from 13 patients who developed ICI-induced diabetes mellitus (ICI-DM) showed significant overexpression of ORM1, PLG, G6PC and a missense mutation in NLRC5. The researcher proposed that NLRC5 mutation could be used to predict ICI-DM (164). The analysis of protein-protein interactions also helped to identify biomarkers, for which four immunogenetic variants were identified by genotyping 39 variants in 18 genes using a multiplex genotyping assay (165). Single-cells RNA sequencing revealed that patients with distinct T cells populations at baseline were under the risk of distinct types of irAEs and this could serve as biomarker for those irAEs. Fewer CD8^+^ T_CM_ cells, more Th2 and Th17 cells were observed in patients with irAEs, and were associated with a higher risk of ICIs-induced arthritis, pneumonitis, and thyroiditis (166). Higher resolution human leucocyte antigen (HLA)-I typing on 179 patients with NSCLC treated with anti-PD-1/PD-L1 found that homozygosity at one or more HLA-I loci was associated with a reduced risk of irAE, including pruritus and rash (relative risk (RR) = 0.61, 95% CI 0.33 − 0.95, *P* = 0.035) and this could also serve as a biomarker (167). A variation in HLA-DRB1 was found to be associated with one or more types of cirAEs, and a more detailed association between HLA-DRB1*11:01 and pruritus was validated (168). This finding was in line with that of a previous study confirming an association between HLA-DRB1*11:01 and atopic dermatitis (AD) (169). The results of 16S rRNA gene amplification and multi-parallel sequencing also indicated that microbiota may serve as a potential biomarker (93, 104). Non-coding RNA (e.g., miR-146a) was found to be associated with irAEs in preclinical studies, and the predictive efficacy was validated by analyzing the effect of a SNP in the *MIR164A* gene on irAEs in 167 patients treated with ICIs. SNP rs2910164 led to reduced miR-164a expression and was associated with an increased risk of irAEs (170). Taken together, these studies via multi-omics technology have shed light on the discovery of biomarkers that can predict irAEs, although the efficacy should be validated using large number of clinical trials and the testing methods must be improved to be suitable for clinical practice.

There’re also many biomarkers for the responsiveness to ICIs therapy being identified by means of multi-omics, with a potential role in predicting irAEs as well. In a rich resource of scRNA-seq and bulk mRNA-seq analysis, B cells and T follicular helper cells were found mediating the response to ICI in breast cancers, and a new predictive gene signature was identified (171). Based on the antigenicity of tumors, the analysis of self-immunopeptidome also served to predict the response of ICIs. This method calculated the ratio of nonsynonymous to synonymous mutation (dN/dS) to discriminate the ‘escaped tumors’ and ‘edited tumors’, with the former presenting neoantigens but escaping immune attack by immunosuppressive mechanisms such as over-expressing PD-L1, thus responsive to ICIs and under risk of irAEs; and the latter escaping by neoantigen-depletion that prevent tumors from being recognized by immune system, thus non-responsive to ICIs (172). A study of proteomics also identified leukemia inhibitory factor (LIF) as a novel predictive biomarker of resistance of ICIs (173). Moreover, a quantitative functional proteomics analysis[QF-Pro] found that functional engagement of the PD-1/PD-L1 complex but not PD-L1 expression alone is highly predictive to the response to ICIs in non-small-cell lung cancer (174). Another multi-omics study of 108 human papilloma virus (HPV)-negative head and neck squamous cell carcinomas (HNSCCs) identified three subtypes with responsiveness to CDK inhibitors, anti-EGFR antibody and immunotherapy, respectively, and an immune-proteogenomic analysis uncovered the mechanisms of immunosuppression and ICIs-resistance (175). Metabolomics studies found that hypoxanthine and histidine in early on-treatment serum (176), indoleamine-2,3-dioxygenase (IDO) (177) and very long-chain fatty acid-containing lipids (VLCFA-containing lipids) (178) were also predictive biomarkers for the response to ICIs. Still, these biomarkers need further validation for the predictive efficacy of irAEs, in addition to the anti-tumor efficacy.

Another important method is radiomics, which assess tumors based on the abundant images from computed tomography (CT), magnetic resonance imaging (MRI) and positron emission tomography/computed tomography (PET/CT) (173, 174). The analysis of genomics, transcriptomics, proteomics and metabolomics, as previously discussed, need puncture or surgery for biopsy. Due to the spatial and temporal heterogeneity, however, it is not available to get whole information by sampling (175). As a widely used and non-invasive diagnosis method, radiographic examination serves as a repeatable and rapid approach for assessing the tumors. These traditional radiographic imaging, equipped with modern technologies such as high-resolution imaging, data mining algorithms and high-throughput analysis, provides global information about the biology of tumors and contributes to predicting the efficacy and adverse events of immunotherapy. Nowadays, radiomics studies seek for biomarkers mainly by analyzing the characteristics of tumor microenvironment (TME) (176), such as tumor-infiltrating lymphocytes (TIL), microcirculation, various signal molecules and extracellular matrix, tightly associated with immunotherapy. As known, TIL is a significant parameter to predict the response to immunotherapy, CT, PET/CT and MRI-based biomarkers to assess TIL have been identified by radiomics studies (4, 177, 178). There’re also radiomics-based prediction models for assessing the PD-L1 expression being proposed, using the radiomics feature combined with clinical characteristics (5–9). Radiomics features that predict ICI-induced pneumonitis were identified by maximum relevance and minimum redundancy feature selection method, anomaly detection algorithm, and leave-one-out cross-validation, despite the predictive efficacy to other types of irAEs remaining unvalidated. Nevertheless, radiomics analysis, involving in the technology of multi-omics, will play an increasingly crucial role in the research of tumors.

## 5 Summary

In this review we discussed the application of ICI therapy to cancers and its adverse events in epidemiology, followed by a detailed discussion of its immunological mechanism and prediction. Cutaneous irAEs represents one of the most common types of irAE associated with ICI therapy and can lead to substantial suffering, as well as hinder the normal application of ICI, with a blockade of the normal role of checkpoints in the regulation of immune response being an initiating factor of irAEs. The immune response is a double-edge sword in that an appropriate immune response can serve as a defense against invading pathogenic microbes, eliminating damaged and aging cells and surveilling the oncogenic cells, whereas a disordered immune response will induce harmful effects. Checkpoints, including positive and negative checkpoints, play a crucial role in limiting the immune response and mediating immune tolerance. In this manner, these checkpoints contribute substantially to the maintenance of immune homeostasis, although it might be used by tumors to suppress immunity. From this perspective, ICIs may induce the non-specific enhancement of immune activity, including both increased cytotoxicity and broadened immune targets, thereby giving rise to an over-activated immune response. Such overactivation can manifest as irAE, and cutaneous irAEs commonly occur early for the distinctive immune signature of the skin as discussed above. For the broad use of ICIs in comprehensive cancer therapy, predicting and identifying irAEs is necessary, especially cirAEs. Previous studies have primarily conducted epidemiological investigations or measured the serum levels of some immune molecules, failing to identify satisfying biomarkers due to the heterogeneity of irAEs. Multi-omics analysis has shed light on the precise mechanism of irAEs and identify several genetic variants, non-coding RNAs and enzymes, which can potentially serve as biomarkers for predicting cirAEs; however, further clinical validation is required. Nevertheless, we believe that multi-omics research will continue to contribute more for both uncovering the mechanism and identifying of biomarkers.

Although various of PD-1/PD-L1 blockers have been applicated in clinical practice, the complete mechanisms of checkpoints such as TIM-3 and LAG-3 still remain unclear and the distinct mechanisms of irAEs induced by different types of ICBs need more researches to elucidate. The clinical manifestations of irAEs also provide a novel pathway to uncover the role of these immune checkpoints in regulating immune homeostasis. More precise understanding of cutaneous immunity and cutaneous immune diseases such as psoriasis and atopic dermatitis is also needed for exploring the mechanisms of cirAEs. The predictive biomarkers for cirAEs will be more precise, specific to certain type and severity of cirAEs. Finally, the developing multi-omics analysis technologies, especially single-cell and spatial multi-omics analysis, will provide more and more information which helps not only to find predictive biomarkers but also to uncover the mechanisms of cirAEs.

## Author contributions

TC conducted the bulk of the writing and the crafting of the figures; XZ performed the formulation of the overarching research goal and the revision of the draft; XZ and XW retrieved literature; YZ critically revised the manuscript. All authors contributed to the article and approved the submitted version.
